# Why osteoarthritis of the knee is called “a wound that does not heal” and why Tai Chi is an effective treatment

**DOI:** 10.3389/fmed.2023.1208326

**Published:** 2023-11-27

**Authors:** Patricia Huston

**Affiliations:** ^1^Department of Family Medicine, Faculty of Medicine, University of Ottawa, Ottawa, ON, Canada; ^2^Institut du Savoir Montfort (Research), University of Ottawa, Ottawa, ON, Canada

**Keywords:** Tai Chi, osteoarthritis, biomechanics, alignment, chronic low-grade inflammation, fibrosis, macrophages, innate immunity

## Abstract

**Context:**

Osteoarthritis (OA) of the knee is common and is associated with other chronic diseases and early mortality. OA is often described as a “wound that does not heal” because a local innate immune response gets dysregulated. Tai Chi is an aerobic mind-body practice that is recommended in national and international clinical practice guidelines as a treatment for OA of the knee. This review addressed two questions: What causes immune dysregulation in the knee? and Why is Tai Chi an effective treatment?

**Recent findings:**

There is now a good understanding of what causes OA of the knee at the cellular level. OA begins in the synovium from a phenotypic shift in synovial macrophages in response to tissue damage. The synovial macrophages release inflammatory cytokines, as part of the first phase of the normal healing and repair process. Cytokines communicate to other cells that there has been damage. This stimulates chondrocytes, osteoblasts, and fibroblasts to release inflammatory cytokines as well. When tissue damage is repetitive, there is repetitive release of inflammatory cytokines, and the normal healing process stops. The most common cause of tissue damage is from abnormal biomechanical forces on the knee that arise from trauma, injury, and misalignment. Tissue damage is made worse when there is systemic low-grade inflammation associated with other chronic conditions. Pain and stiffness often result in decreased physical activity, which leads to muscle weakness, progressive instability of the joint, and an increased risk of falls, further injuring the knee. Tai Chi improves alignment, optimizes the biomechanical forces on the knee, strengthens the lower limbs, and decreases systemic inflammation. Tai Chi improves balance and decreases the risk of falls and further injury. There is clinical and experimental evidence to suggest that by removing the causes of cell dysregulation, Tai Chi enables the normal healing and repair process to resume.

**Conclusion:**

Knee OA is a wound that does not heal primarily because repetitive adverse forces on the knee cause synovial macrophages and then local chondrocytes, osteocytes and fibroblasts to dysregulate and stop the normal healing and repair process. Tai Chi mitigates adverse forces on the knee and stabilizes the joint, creating the conditions whereby the normal healing and repair process can resume. Further research is needed.

## Introduction

Arthritis is common. In the United States, almost one in four adults develop arthritis and it is the number one cause of work-related disability (1). Osteoarthritis (OA) is the most common form of arthritis. Compared to those without OA, people with OA are more sedentary, have more comorbidities (2), and have a 20% higher age-adjusted mortality rate (3, 4).

The understanding of OA and its recommended treatment has changed in the last 15 years. Traditionally OA was thought to arise from “normal wear and tear” of the cartilage with age, and the treatment was to rest the affected joint and take pain medications until a knee replacement could be offered. The current understanding of OA as “a wound that does not heal” was first identified in 2008. At that time, synovial inflammation had been increasingly recognized as an important process in OA pathology, but what caused it and how it was linked to cartilage loss were not well-defined. Scanzello and colleagues proposed that synovitis in the knee was caused by a persistent innate immune response leading to acute inflammation (5). Further research revealed this immune response is part of the normal healing and repair process but when it becomes dysregulated, it causes chronic inflammation and damage (6–10).

Since that time, treatment recommendations have gone from rest to aerobic activity, strength training and normalizing weight as first line therapies for OA of the knee (11–14). In light of both the new understanding that the innate immune system is involved in OA, and the absence of effective pharmacotherapies to address the underlying pathology of OA, there have been calls to “develop innovative ideas and approaches that go beyond conventional paradigms” (15).

Tai Chi is an aerobic mind–body practice that, based on systematic reviews of multiple randomized controlled trials (RCTs), is very effective for OA of the knee (16, 17). For example, in two head-to-head RCTs comparing Tai Chi with physiotherapy, both resulted in similar improvements of pain and function (18, 19). One trial documented a clear dose–response relationship. In both the Tai Chi and physiotherapy groups, median response time was 2 weeks for ≥20% improvement in pain and function and 4–5 weeks for ≥50% improvement (19). Tai Chi is now recommended for the treatment of OA of the knee in international guidelines (11) as well as national guidelines in the United States (12), and Canada (20). Tai Chi can begin before a child starts to attend school to 80 years and older. It involves slow movements, requires no special equipment, and is appropriate for those who may have lost their former level of fitness.

The goal of this article is to answer two questions: What causes immune dysregulation in the knee? and Why is Tai Chi an effective treatment?

## Normal and dysregulated tissue repair

Some of the greatest gains in understanding knee OA pathology have arisen from the discovery that the immune cells in the knee are also involved in the normal healing and repair process; it is only when these cells are dysregulated does a pathological process ensue. These are complex processes involving multiple immune cells, signaling molecules, enzymes, metabolic processes, and epigenetics. The following description focuses on the main cells of the knee: synovial cells, cartilage and bone cells, as well as connective tissue and fat cells, and how they all interact in the process of repair as well as pathology.

### Resident macrophages orchestrate normal tissue repair

Throughout the body, response to injury and hypoxia is orchestrated by resident macrophages, which are a part of the innate immune system (21). Unlike circulating macrophages, resident macrophages arise in all tissues of the body during embryonic development and remain there for the entire lifespan (22).

Resident macrophages are the body’s first line of defense for threats and tissue damage of all types whether be it from infection, a wound, ischemia or injury (23). Throughout the body, resident macrophages undergo phenotypic shifts depending on local circumstances (24). In their steady-state, these macrophages are in a surveillance phenotype (called M0).

When macrophages discover tissue damage, they transition into an inflammatory phenotype (M1) and release pro-inflammatory cytokines (21). Cytokines are signaling molecules that communicate to other cells that there has been damage. Cytokines can attract other immune cells to help remove dead tissue and extravasated blood cells from the area. Once the “clean up” is complete, macrophages transition to their anti-inflammatory phenotype (M2), and release anti-inflammatory cytokines to facilitate tissue repair.

Resident macrophages are called by different names in different tissues. They are called microglia in the brain (25), Kupffer cells in the liver (26), alveolar macrophages in the lungs (27), and renal macrophages in the kidney (28). In the knee joint, resident macrophages are called synovial macrophages or synoviocytes A and B (29).

Synovial macrophages are also central to OA progression (30). How does a cell that orchestrates the normal healing and repair process cause disease?

### Osteoarthritis arises from macrophage dysregulation

There are four stages in OA cellular pathology. The first stage is cytokine release by synovial macrophages in the synovial fluid. This is stimulated by micro cartilage fragments and an endogenous molecule called damage-associated molecular patterns (DAMPs) that are released in response to adverse biomechanical forces on the knee (31). Micro fragments and DAMPS are what stimulate synovial macrophages to shift into their M1 phenotype and release inflammatory cytokines (32). Cytokines in the synovial fluid lead to synovial inflammation, which persists throughout all stages of OA of the knee (33). This may help to explain why in the early stages of OA no radiological changes are seen. OA starts deep in the knee joint. It is only well into the second stage when the cartilage thins enough that the pathological changes of OA can be seen radiologically.

The second stage of OA of the knee is cytokine release by chondrocytes in the cartilage. Normal turnover of the cartilage is managed by chondrocytes. When synovial macrophages release inflammatory cytokines it signals to chondrocytes there has been tissue damage via a process called intracellular cross-talk (34). The inflammatory cytokines stimulate chondrocytes to switch from an anabolic to a catabolic phenotype which then start to release their own inflammatory cytokines. Ongoing cytokine release by catabolic chondrocytes stimulate an enzymatic cascade that starts to degrade the cartilage matrix in a feed-forward loop (35).

The third stage of OA of the knee is cytokine release by osteocytes. As cartilage erodes, it can no longer equalize mechanical forces on the bone. When osteocytes are exposed to inflammatory cytokines, they start to release their own inflammatory cytokines. This stimulates a phenotypic shift in osteocytes toward osteoclast activity, resulting in subchondral bone absorption (36). Bone erosion further exacerbates the adverse mechanical load on the joint.

The fourth stage is cytokine release by fibroblasts and fat cells in the surrounding tissues and can begin during the other stages. Fibroblasts are interstitial connective tissue cells that are found in all tissues, including the synovium and infrapatellar fat pad, and are the main connective tissue cell found in tendons and ligaments. When exposed to inflammatory cytokines, fibroblasts release their own cytokines and cause damage. For example, synovial fibroblasts in the synovial membrane, release cytokines that increase synovitis, and extracellular matrix that leads to stiffness and eventually synovial fibrosis (37, 38). The increased stiffness, in turn, stimulates synovial macrophages into a new M1 mode, setting up a positive feedback loop of inflammation (35).

The infrapatellar fat pad is functionally linked with the synovial membrane (39). Macrophages are abundant in adipose tissue and respond to inflammatory cytokines released by synovial fibroblasts by releasing their own inflammatory cytokines called adipokines (40). This, in turn, stimulates fibrocytes within the collagen stroma to release increased matrix associated with thickening of the interlobular septa (41). Adipokines in the infrapatellar fat pad have been found to enter into the synovial fluid and accelerate the cellular senescence of chondrocytes (31) and may further contribute to synovial fibrosis (42).

Recurrent abnormal loading of the meniscus and anterior cruciate ligament is a mechanical stimuli for fibroblast dysregulation and, if chronic, will lead to chronic inflammation and progressive fibrosis of the ligaments (43).

**Figure 1:**
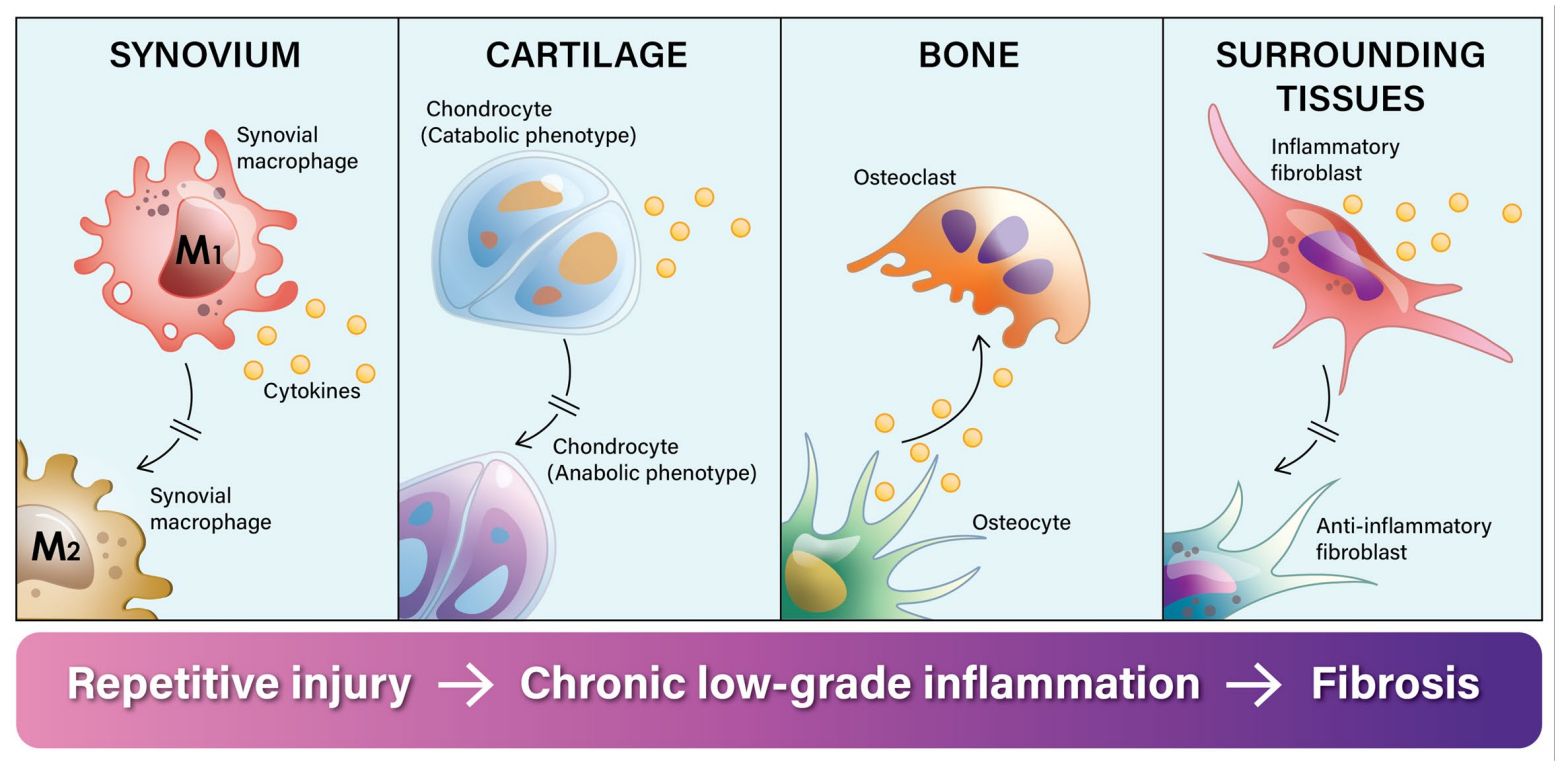
Knee osteoarthritis is called a “a wound that does not heal” because the normal tissue healing and repair process gets stalled in the inflammatory phase. Chronic adverse biomechanical forces on the knee joint disrupt and dysregulate the normal repair cycle of tissues in the knee by repeatedly stimulating synovial macrophages to transition into their inflammatory phenotype (M1), which prevents them from going into their anti-inflammatory phenotype (M2) and repairing the tissue. In response to injury and micro-fragments in the synovial fluid, synovial macrophages release inflammatory cytokines. Through a process of intracellular cross-talk, this inflammatory process spreads. First it stimulates chondrocytes to switch from an anabolic phenotype to a predominantly catabolic phenotype, leading to cartilage erosion. Inflammatory cytokines stimulate a phenotypic shift in osteoblasts toward osteoclast activity and leads to subchondral bone absorption. This progressively erodes the bone, and the inflammatory process further spreads to the surrounding fat pad and connective tissue. Intracellular cross-talk stimulates fibroblasts into an inflammatory phenotype and the release of inflammatory cytokines as well as excess extra-cellular matrix. The end result of this dysregulated process is destruction of the joint and surrounding fibrosis.

In summary, adverse biomechanical forces on the joint lead to progressive changes in the cellular phenotypes of all the tissue cells of the joint. When this occurs repetitively, chronic inflammation ensues. Chronic inflammation prevents the different cells from going into their M2 anti-inflammatory phenotype, which is needed to complete the healing and repair process. This dysregulated cycle of inflammation is the major factor that explains why OA is called “a wound that does not heal” (Figure 1).

## What causes immune dysregulation in the knee?

Traditionally, the usual risk factors for OA were identified as increasing age, being a woman, obesity, injuries, bone deformities, genetics and some metabolic diseases, such as diabetes (44, 45). It was then realized that most of these risk factors—obesity, acute and repeated stress injuries, and bone deformities—all result in adverse or abnormal forces on the knee.

Similar to coronary artery disease, physical activity can be either an OA prevention strategy or a precipitating factor for OA progression. When done within someone’s physiologic and biomechanical limits, it has beneficial effects. But when it exceeds someone’s physiologic/biomechanical limits, it can do harm. Specifically, physical activities that involve normal physiologic loads on the knee promote cartilage anabolism, but traumatic or hyper-physiologic loads trigger cartilage catabolism (46, 47).

A recent meta-analysis established that adverse biomechanics were associated with an increased risk of OA in over 90% of studies (8). In addition, systemic low-grade inflammation and other exacerbating factors also contribute to arthritic changes. So, what are the sources of adverse biomechanics, systemic low-grade inflammation, and other exacerbating factors?

### Adverse biomechanical forces

Trauma is a common cause of adverse biomechanical forces on the knee, which can be acute (caused by falls, sports injuries, and motor vehicle accidents) or chronic, arising from repetitive low-grade trauma to the knee. A past history of knee injury in young adults is associated with a 6-fold increased risk of subsequent OA of the knee (48, 49). Knee injuries commonly involve the anterior cruciate ligament, the meniscus, or both (50–52). Repetitive or overuse injuries often arise from common activities, such as running and bicycling (53, 54), or may be linked to an underlying musculoskeletal deformity that repetitive use reveals, such as varus and valgus deformities (55). Patello-femoral syndrome is common in adolescents (56) and is associated with OA in adulthood (57). Patello-femoral syndrome has recently been linked with deficits in hip abduction, extension and external rotation, which are also associated with OA of the knee (58–60).

Misalignment of the ankle is related to OA of the knee (61), especially when the foot is pronated (62). This makes sense when one considers the role of the foot in receiving and distributing the ground reaction force that arises when the foot strikes the ground. Mechanical loading from increased ground reaction force directed to the knee has been implicated in the pathogenesis of OA of the knee (63). The use of high-heeled shoes has clearly been associated with OA of the knee (64) and may explain why OA is more common in women.

### Increased systemic inflammation

It is thought that systemic low-grade inflammation—that is characterized as increased levels of inflammatory cytokines circulating in the bloodstream—either exacerbates OA or decreases the local threshold for its development (65). Obesity is a common source of systemic low-grade inflammation (66). This occurs through the release of adipokines—a type of pro-inflammatory cytokine—that enter the bloodstream. Circulating adipokines have been directly linked to disrupting cartilage homeostasis (67). A systematic review of prospective studies found the risk of knee OA increases by 35% with every 5  kg/m^2^ increase in BMI (68).

Systemic low-grade inflammation was initially associated with aging (69), but now it is more clearly associated with chronic disease, including diabetes (70), heart disease (71), chronic lung disease (72), and more. In a recent international study, almost two thirds of people with knee OA (62%) had at least one co-morbidity, with hypertension, heart disease and diabetes being the most common (73).

Chronic disease progression has also been associated with social isolation, which often occurs among older adults with pain and decreased mobility (74). The adverse effects of stress and social isolation appear to be mediated through a chronic sympathetic response, which in turn amplifies systemic low-grade inflammation (75, 76). Thus, as people age with OA of the knee, multiple factors conspire to increase both local and systemic inflammation and advance osteoarthritic pathology.

### Exacerbating factors

There are several exacerbating factors that compound adverse forces on the knee: obesity, aging, sedentariness, and progressive muscle weakness. Over 70% of Americans are either overweight or obese (77). Obesity is a well-known risk factor for OA of the knee (11–13, 78), in part because the increased weight alters the biomechanical forces on the knee (79, 80).

People with knee OA often decrease their physical activity because of pain, leading to progressive quadricep weakness, which destabilizes the joint and is linked to a loss of proprioception (81). A recent systematic review concluded that quadriceps weakness was a better predictor of OA of the knee than joint space narrowing on x-ray (82).

Unfortunately, there is a compounding nature to these factors. A systematic review found that people with OA of the knee were more likely to have misalignment, muscle weakness, joint laxity, and proprioception deficits (53), putting them at an increased risk of falls (83). And falls once again risks trauma to the knee. So, how can Tai Chi change this process?

## Why is Tai Chi an effective treatment?

Tai Chi is an aerobic mind–body practice that involves mindful concentration, a series of biomechanically sound movements, and abdominal breathing. The center of gravity is low. The ankles, knees, and hips are often in flexion, and the foot moves before weight is transferred between the feet. Weight transfers occur through co-contraction of the agonist (movement) muscles of one leg supported by the antagonist (stabilizer) muscles of the other leg. When the body is alignment, and arm and leg movements are synchronized and coordinated with mindful breathing, the body is able to move with precision and ease.

Given that physical activity is now consistently recommended for OA of the knee (11–14, 78), one might conclude that Tai Chi is simply one of many options. However, there are six different types of physical activity that have been recommended for OA of the knee. All current guidelines recommend aerobics and strength training (11, 12, 14, 78, 84), two guidelines mention balance exercises (11, 12) and this may involve exercises to improve gait and postural control (58). And two recent trends are exercises to improve proprioception (85, 86) and neuromuscular training (87, 88).

Current rehabilitation for OA of the knee may involve all six types of exercise. Once rehabilitation is completed, however, keeping up with all of them could be challenging.

Tai Chi is an “all in one” option. It is an aerobic activity (89, 90), that increases lower extremity strength (91–93), balance (91, 92, 94, 95), gait and postural control (96, 97), improves proprioception (98, 99), and has similarities with neuromuscular training (100, 101). And, unlike many sports, Tai Chi has a very low risk of injury (102, 103) (Table 1). Tai Chi is not just another type of physical activity; it is a comprehensive form of physical re-education (104). Tai Chi improves knee OA in three ways.

**Table 1:** Comparison of exercise recommendations for knee osteoarthritis and Tai Chi.

Exercise recommendations for knee osteoarthritis	Tai Chi	
Aerobics (11–14, 78)	Tai Chi is an aerobic activity (89, 90)	
Strength training (11–14, 78)	Tai Chi increases lower limb strength (91–93)	
Balance training (11, 12)	Tai Chi improves balance (91, 94, 95)	
Gait and postural control (58)	Tai Chi improves gait and postural control (96, 97)	
Proprioception exercises (85, 86)	Tai Chi improves proprioception (98, 99)	
Neuromuscular training (87, 88)	Tai Chi has similarities with neuromuscular training (100, 101)	

### Optimal biomechanical forces

Tai Chi fosters optimal alignment of the hip, knee, and ankle joints (105). Good alignment helps to improve balance (94), proprioception (98), as well as gait and postural control (96) especially in the elderly (91).

### Decreased systemic inflammation

Tai Chi is not just about biomechanics. It is also known to decrease systemic inflammation. It is now well-established that elevated serum levels of inflammatory cytokines, such as IL-6 and TNF-α, are associated with knee cartilage loss in older adults (106). A meta-analysis found that Tai Chi significantly reduced serum TNF-α and decreased IL-6 in those who attended most of the Tai Chi classes (107).

Physical activity in general is known to increase myokines, an anti-inflammatory cytokine released from muscle with physical activity, and this can moderate the effects of inflammatory cytokines (108). Tai Chi also increases myokine levels (109).

It is increasingly recognized that the immune system interacts with the autonomic nervous system (110, 111). Exercise is known to dampen the sympathetic response and protect against the upregulation of inflammatory cytokines (112). In addition, mind-body exercises, such as yoga and Tai Chi, are known for their parasympathetic or relaxation response thought to be due to mindfulness and abdominal breathing (113). Tai Chi also mitigates the inflammatory effect of social isolation. Offered in community-based classes, Tai Chi has been shown to decrease social isolation in the elderly (114–116).

### Stabilizing factors

There are other factors that help restore and maintain a healthy knee joint. Two recent systematic reviews have highlighted that Tai Chi improves strength, especially in the lower limbs (91–93) likely through co-contraction of the lower limb muscles with movement. This helps to stabilize the knee and prevent joint laxity. Two biomechanical studies found Tai Chi produces less of a load on the knee joint than walking (100, 101) and improves the plantar load on the feet (117) which is often abnormal in individuals with knee OA (118). Tai Chi is associated with a decreased ground reaction force from gentle weight transfers and an even weight distribution on the feet (119). Tai Chi significantly improves proprioception of the lower limbs (98, 99).

Finally, Tai Chi is well-known to decrease the risk of falls. The evidence has been so compelling, Tai Chi has long been recommended as an effective fall prevention strategy in older adults (120, 121). A decrease in the risk of falls will, in turn, decrease the risk of further trauma to the knee.

**Figure 2:**
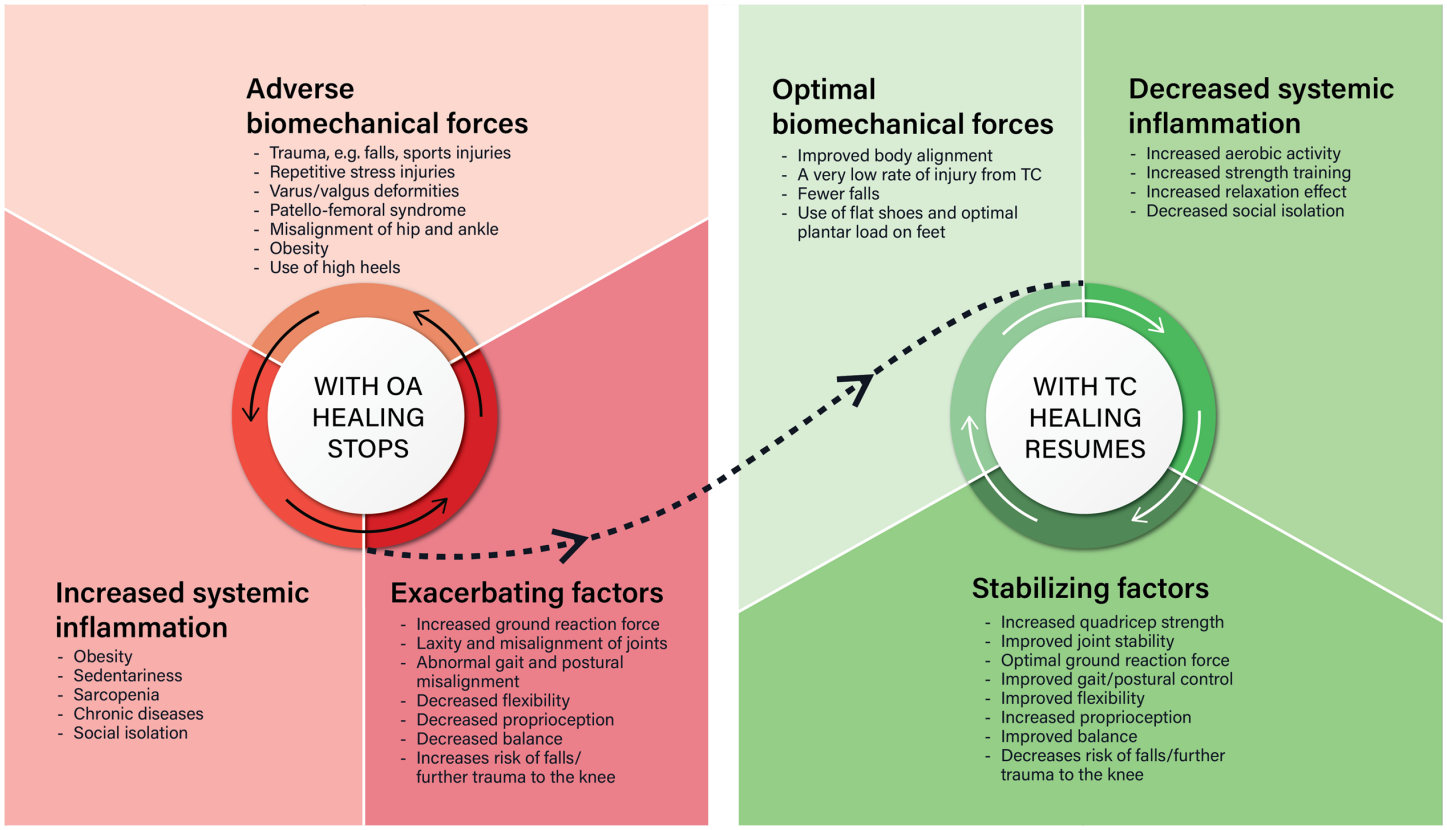
There are striking similarities and differences between what happens in osteoarthritis (OA) and Tai Chi (TC). The similarities are that both are self-perpetuating cycles that involve biomechanical forces on the knee, systemic low-grade inflammation, and other factors. In OA of the knee, adverse biomechanical forces on the knee causes phenotypic shifts in the key cells of the knee, that cause local inflammation which stops the normal tissue repair process. This is made worse by increased systemic low-grade inflammation and other exacerbating factors. Tai Chi improves body alignment, optimizes the biomechanical forces on the knee, decreases systemic inflammation, and stabilizes the knee joint so the normal tissue repair process can resume.

In summary, the pathophysiology of OA of the knee and the therapeutic effects of Tai Chi are both self-perpetuating cycles. With OA of the knee there is a cycle of dysregulated cytokine release arising from repetitive tissue damage which stops the normal healing process. With Tai Chi, conditions are fostered that helps the normal healing process to resume. However, stopping Tai Chi means the cycle could revert again to osteoarthritic progression. These cycles are summarized in Figure 2.

### Tai Chi and cellular phenotypes

Does Tai Chi actually change the phenotype of cells? To date it appears no study has specifically assessed this. It has been suggested that moderate physical activity in general helps to maintain or re-establish the physiological function of synovial macrophages (122). And in an animal study of experimentally induced OA, those who had been physically active beforehand were found to have more anti-inflammatory cytokines reflective of the M2 synovial macrophages needed to complete the repair process (123). It would not be surprising if people with OA of the knee, were found to have more M2 synovial macrophages after taking Tai Chi classes for 8 weeks than before they began—especially if they had improvements in their knee pain and function over that time.

Other evidence to support this idea comes from a recent clinical study of high tibial osteotomies, done to improve biomechanical forces on the knee. Following the procedure, there was less synovial inflammation and more M2 macrophages facilitating the completion of the normal tissue repair cycle (124). Since Tai Chi improves biomechanical forces on the knee, it would be reasonable to conjecture that Tai Chi optimizes cell phenotypes to enable the normal healing process to resume.

## Discussion

This is the first time that Tai Chi as a treatment for OA of the knee has been linked to correcting the conditions that cause immune dysregulation in the knee. The wound does not heal in OA primarily because repetitive adverse biomechanical forces on the knee cause phenotypic shifts in synovial macrophages and other key cells of the joint causing chronic local inflammation that stops the normal healing process. This is made worse if there is systemic inflammation and other exacerbating factors. Tai Chi improves body alignment, strengthens the lower limbs, stabilizes the knee and decreases both local and systemic inflammation, enabling the normal healing and repair process to resume.

There are some limitations to consider. Much more is known on the pathology of OA than described. Other immune cells are involved, there are many phenotypes of macrophages, many cytokines and different enzymatic cascades they evoke, and all this is linked with epigenetic changes. Although the interaction between the immune and autonomic nervous system was briefly described, there are also metabolic, hormonal, and other influences, such as mitochondrial dysfunction and oxidative stress, that have a role in OA pathology.

Likewise, although a lot is known on the therapeutic effects of Tai Chi for OA of the knee, Tai Chi has many other therapeutic effects that were not described here (125). And Tai Chi has other mechanisms of action, such as increasing the functional connectivity of the brain (126). Tai Chi may well have additional mechanisms of action that have yet to be discovered.

Clearly, more research is needed. To date, research on the phenotypic shifts of cells in knee OA has focused on the potential to develop new therapeutic interventions. There have been calls to “develop a drug that skews inflammation toward a pro-chondrogenic microenvironment” (127) and stem cell transplants are still under evaluation for their potential to increase chondrocytes and improve cartilage recovery (128, 129). However, the phenotypic effects of normalizing weight, getting regular physical activity and optimizing biomechanical forces on the knee should not be overlooked. It is likely that not only Tai Chi, but any aerobic exercise (that is biomechanically sound) will optimize the phenotypes of cells in the knee. Research assessing the relative importance of strength training and other exercise types is also indicated.

There are clinical implications to consider. For example, when OA patients come to get physician clearance to start a new exercise program, it would be useful to have a summary of the adverse biomechanical forces on the knee for different types of sports and physical activities (130). And consideration could be given to recommending a biomechanical assessment of the knee (131).

One of the most positive implications of this new understanding for knee OA, is that osteoarthritis is starting to be seen as a reversible disease. OA can be reversed if there is intervention early in the course of disease (32). Understanding what is needed to re-establish the normal healing and repair process in knee OA may be a new motivation for lifestyle change. Much like cardiac rehabilitation supports lifestlye change for those with heart disease, secondary prevention programs could support lifestyle change for those with OA. This would include biomechanical assessment, education, coaching and progressive patient self-management that is well-linked with both clinical care and exercise classes in the community (132, 133). OA of the knee is a common reason for older people to become sedentary. Recent international physical activity guidelines for older adults identify the importance of people regaining and maintaining regular physical activity: it can mitigate most chronic diseases, improve mental health and quality of life, and prevent premature mortality (134).

### Conclusion

Synovial macrophages are key to understanding both knee OA pathology and the effectiveness of Tai Chi. Under adverse biomechanical conditions, synovial macrophages become dysregulated and this dysregulation spreads to the other cells in the joint and leads to OA pathology. When local biomechanical conditions are optimized with an activity such as Tai Chi, synovial macrophages can resume orchestrating the normal healing process. More research is needed on different types of exercise for knee OA and on how to help people make lifestyle changes more effectively. Some clinicians have hesitated to prescribe Tai Chi as it is a mind-body practice whose therapeutic effects are not yet widely known (135). However, mind-body practices are increasingly mainstream (136) and, based on RCTs, guideline recommendations and mechanism of action studies, clinicians can now be confident in recommending Tai Chi so that the wound of knee OA can heal.

## Author contributions

The author confirms being the sole contributor of this work and has approved it for publication.
